# Supplementary material for: Engineering of Humanized PSMA-Directed CAR T Cells for Potent and Specific Elimination of Prostate Cancer Cells
Source: Cells. 2025 Aug 28;14(17):1333. doi: 10.3390/cells14171333 (PMC12428541; doi:10.3390/cells14171333)
Supplement: Supplementary file 1 [file cells-14-01333-s001.zip › cells-3826178-supplementary.pdf]

cells

MDPI

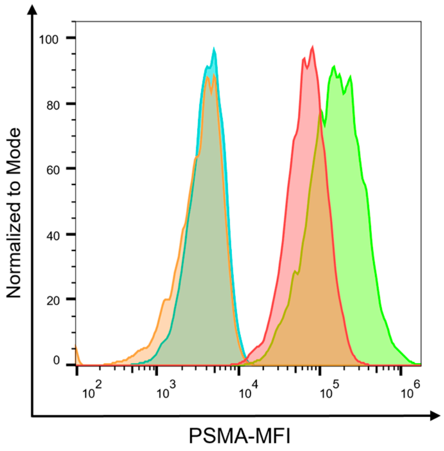

Cell Line	MFI
C4-2	78657
PC3-PSMA	199842
DU145	3947
PC3	4490

Supplementary Figure S1 | PSMA expression patterns on target cells. Included are the PSMA+ prostate cancer cell lines C4-2 and PC3-PSMA, as well as the PSMA- cell lines PC3 and DU145. PC3-PSMA is originally generated by viral transduction of the PSMA-negative cell line PC3, here included as a control. PC3-PSMA cells show the overall highest PSMA expression.

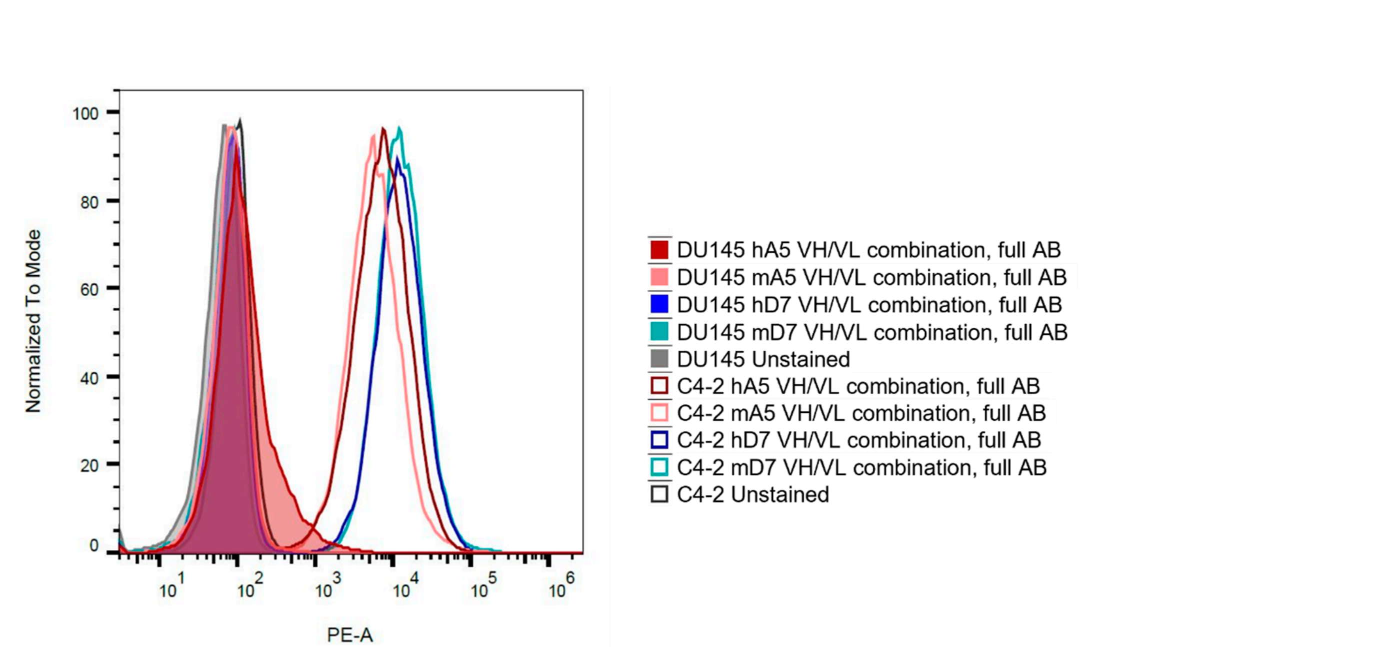

Supplementary Figure S2 | Binding of the VH/VL combinations in hIgG1 format to the PSMA-negative cell line DU145 (shaded) and PSMA-positive cell line C4-2 (outline). Curves are normalized to the mode. The antibodies are detected using a goat anti-human Ig (H + L) antibody conjugated to R-phycoerythin (Southern Biotech). Mean fluorescence intensity (MFI) values of stained cells are acquired using a FACSymphony A1 flow cytometer and analyzed with FlowJo software. Abbreviations: AB – antibody, mD7 – murine D7 VH/VL combination; hD7 – humanized D7 VH/VL combination; mA5 – murine A5 VH/VL combination; and hA5 – humanized A5 VH/VL combination.
